# RAGE, Receptor of Advanced Glycation Endoproducts, Negatively Regulates Chondrocytes Differentiation

**DOI:** 10.1371/journal.pone.0108819

**Published:** 2014-10-02

**Authors:** Tatsuya Kosaka, Rino Fukui, Mio Matsui, Yuko Kurosaka, Haruka Nishimura, Motoki Tanabe, Yuuki Takakura, Keisuke Iwai, Takuya Waki, Takashi Fujita

**Affiliations:** Molecular Toxicology lab, Department of Pharmaceutical Sciences, Ritsumeikan University, Shiga, Japan; University of Miami, United States of America

## Abstract

RAGE, receptor for advanced glycation endoproducts (AGE), has been characterized as an activator of osteoclastgenesis. However, whether RAGE directly regulates chondrocyte proliferation and differentiation is unclear. Here, we show that RAGE has an inhibitory role in chondrocyte differentiation. RAGE expression was observed in chondrocytes from the prehypertrophic to hypertrophic regions. In cultured cells, overexpression of *RAGE* or dominant-negative-*RAGE* (DN-*RAGE*) demonstrated that *RAGE* inhibited cartilaginous matrix production, while DN-*RAGE* promoted production. Additionally, *RAGE* regulated Ihh and Col10a1 negatively but upregulated PTHrP receptor. Ihh promoter analysis and real-time PCR analysis suggested that downregulation of Cdxs was the key for *RAGE*-induced inhibition of chondrocyte differentiation. Overexpression of the NF-κB inhibitor *I-κB-SR* inhibited *RAGE*-induced NF-*κ*B activation, but did not influence inhibition of cartilaginous matrix production by *RAGE*. The inhibitory action of *RAGE* was restored by the Rho family GTPases inhibitor Toxin B. Furthermore, inhibitory action on Ihh, Col10a1 and Cdxs was reproduced by constitutively active forms, *L63RhoA*, *L61Rac*, and *L61Cdc42*, but not by *I-κB-SR*. *Cdx1* induced Ihh and Col10a1 expressions and directly interacted with Ihh promoter. Retinoic acid (RA) partially rescued the inhibitory action of *RAGE*. These data combined suggests that RAGE negatively regulates chondrocyte differentiation at the prehypertrophic stage by modulating NF-*κ*B-independent and Rho family GTPases-dependent mechanisms.

## Introduction

Advanced glycation end products (AGEs) are permanently modified protein derivatives formed in the presence of reducing sugars, such as glucose and fructose by non-enzymatic glycation, oxidation and dehydration reactions [1]. In diabetic vascular complications including bone disease, AGEs are known to accumulate in various tissues at an extremely accelerated rate [2], [3]. 3 classes of AGE receptors: RAGE (receptor for AGE), a complex of OST-48/80KH/galectin-3, class A scavenger receptor have been identified [4]–[8]. RAGE is a type I transmembrane protein belonging to the immunoglobulin superfamily and is composed of an extracellular region, a hydrophobic transmembrane-spanning domain and a short cytoplasmic tail [5]. Deletion of the cytoplasmic domain of RAGE imparts a dominant negative (DN)-effect on RAGE-dependent activation of cell signaling both *in vitro* and *in vivo* [9]–[11]. RAGE signaling axes ultimately induce the nuclear translocation of Nuclear factor (NF)-κB, a hallmark of the pro-inflammatory signal transduction cascade [12]. RAGE is involved in a broad range of inflammatory, degenerative and hyper proliferative diseases, including sepsis, rheumatoid arthritis, diabetic nephropathy/angiopathy, atherosclerosis, cancer and neurological disorders [13], [14].

RAGE is expressed in a range of cell types, including smooth muscle cells, fibroblasts, osteoblasts, and osteoclasts [15]–[18]. Recent studies using the knockout strategy demonstrated that RAGE, via regulating osteoclast maturation and activation, acts as a bone modulator [15], [19]. RAGE null mice showed following phenotypes: increased bone mass and bone mineral density, enhanced bone biomechanical strength and decreased osteoclastic bone resorptive activity. Osteoclasts from RAGE null mice exhibited disrupted actin ring and sealing zone structures, impaired differentiation and attenuated bone resorption activity [15]. Osteoclasts are regulated by bone-forming cells such as osteoblasts and stromal cells. In hard tissue, the accumulation of AGEs by crosslinking in collagen fibrils contributes to disturbed bone modeling and deterioration of bone tissue quality [20]. AGEs-dependent fragility of the bone alters bone mechanical properties such as stiffness and strength [21]. In addition, RAGE is also expressed in articular chondrocytes and it may mediate AGEs-induced osteoarthritis [22]. In the human articular cartilage, an increase in AGE levels negatively affects the proteoglycan synthesis, thereby reducing cellular turnover and repair capacity in turn contributing to the degradation of tissue [23]. These observations on skeletalgenesis led us to the hypothesis that RAGE might directly modulate chondrocyte functions such as proliferation or differentiation.

With respect to RAGE signaling, several target genes have been identified in the past, including proinflammetory mediators, matrix mataroproteinases and adhesion molecules. However, their expression critically depends on cell type, microenvironment and quality of the stimulus [24]. Additionally, although multiple intracellular signaling pathways, including MAP kinases, Rho GTPases, PI3K, JAK/STAT, and NF-κB, have been found to be altered following RAGE stimulation, the molecular mechanisms on how RAGE triggers intracellular signaling to regulate cellular decisions remain largely elusive and the identity of direct signaling molecules downstream of the receptor to modulate chondrocytes are still unknown [25]–[27].

During early skeletal development, mesenchymal cells condense and acquire the chondrocyte phenotype including ability to produce Col2a1 and proteoglycan. In the process of endochondral ossification, immature chondrocytes proliferate and chondrocytes at the center of the cartilaginous skeleton begin to mature to become prehypertrophic chondrocytes which express parathyroid hormone/parathyroid hormone-related peptide receptor (PTHrP-R) and Indian hedgehog (Ihh). The prehypertrophic chondrocytes further mature to hypertrophic chondrocytes that express Col10a1. Upon the terminal differentiation terminal hypertrophic chondrocytes express osteopontin, the matrix is mineralized, vascular vessels invade the calcified cartilage and finally the cartilage is replaced by bone. Chondrocyte proliferation and differentiation occur in an organized manner and result in the formation of a growth plate that is composed of layers of chondrocytes at different stages of differentiation, including resting, proliferating, prehypertrophic, hypertrophic and terminal hypertrophic chondrocytes [28]. However, it is not clear how RAGE influences chondrocyte maturation.

Existing data suggests that accumulated AGE predisposes development of metabolic bone diseases such as osteoarthritis, rheumatoid arthritis. In order to clarify the precise role of AGE-RAGE signaling axis, we studied effects of overexpression of *RAGE* or DN-*RAGE* on proliferation, matrix synthesis and differentiation in chondrocytes. Here, we report that RAGE negatively mediated chondrocyte differentiation at prehypertrophic stage thorough NF-κB-independent and Rho family GTPases-dependent mechanisms.

## Materials and Methods

### Cell cultures and retroviruses

ATDC5, MC3T3-E1 cells were purchased from RIKEN Cell Bank (Tsukuba Science City, Japan) and cultured as described previously [29]. To produce the retrovirus, Plat-E cells were transfected with pMXs-neo-derived vectors [30] by Fugene 6 (Roche Diagnostics, Tokyo, Japan). For infection, cells were incubated in the culture supernatant of Plat-E transfectants with 4 µg/ml polybrene for 24 hours and then selected using antibiotic G418 (400 µg/ml). Adenoviruses amplified in 293 cells described below were used as a supernatant aliquot after centrifugation and stored at −85°C until use. NF-κB inhibitor *I-κB-SR* adenovirus, which has alanine substitutions at serines 32 and 36 was gifted from Dr. Jun-ichiro Ionue [31]. Toxin B and HMGB1 were purchased from Sigma (Sigma Chemical Co., Saint Luis, MO). Primary chondrocytes were prepared as described previously [32]. Briefly, isolated skeleton from E13.5 embryo was dispersed by PBS containing 0.1% trypsin/0.1% collagenase. The protocol used here meets the guideline of the Japanese Society for Pharmacology and was approved by the Committee for Ethical Use of Experimental Animals at Ritsumeikan University, permit number (BKC2010-3-3, BKC2013-018).

### Immunocytochemical and immunohistochemical analysis

Detection of alkaline phosphatase activity was performed as described previously [29]. For immunocytochemical analysis, antibodies were reacted before fixation. Cell cultured in chamber slides were reacted with RAGE antibody (AB9714; 1∶1000, Millipore, Billerica, MA), and washed by PBS, then fixed by 10% neutralized formaldehyde. Then, samples were reacted with FITC-conjugated anti-rabbit antibody and 1 µg/ml DAPI (4′, 6-diamidino-2-phenylindole dihydrochloride). For histological analyses, legs of embryos at E15.5 were fixed in 4% paraformaldehyde/phosphate buffered saline, and the limb was embedded in paraffin as described previously and several modifications [29]. Sections (7 µm thick) were subjected to immunohistochemical analysis using antibodies of anti-RAGE (1∶500) or anti-Cdx1 (ab116111; 1∶500, Abcam, Tokyo, Japan). Antibodies reactions were performed in Blocking One Histo (Nacalai Tesque, Kyoto, Japan). Fluorescence images were acquired using EVOS FL cell imaging system (Life Technologies Corp., Tokyo, Japan). Transmit and green mode images were overlaid and obtained merged images.

### Immunoblot

Immunoblot analysis was performed as described previously [29]. Proteins were resolved by SDS-10% polyacrylamide gel electrophoresis. The blots were first incubated with rabbit anti-RAGE(1∶2000), rabbit anti-Actin (I-19; 1∶2000, Santa Cruz Biotechnology, Inc., Santa Cruz, CA), mouse anti-Myc (9E10) (sc-40; 1∶1000, Santa Cruz Biotechnology, Inc.), rabbit anti-Cdx1 antibody, and then reacted with horseradish peroxidase-conjugated anti-rabbit IgG, anti-mouse IgG (Cell Signaling Tech., Beverly, MA), or anti-goat IgG (Santa Cruz Biotechnology, Inc.) (second antibody dilution, 1∶1000). All antibodies reactions were performed in Blocking one (Nacalai Tesque). Chemiluminescence signals were obtained from reaction with Chemi Lumi One Plus Reagent (Nacalai Tesque), and monitored by LAS4000 system (FUJI film, Tokyo, Japan). All images were obtained within 5-min in adequate mode.

### AGE preparation

AGE preparation conducted as described by Tanaka et al. [33]. 50 mg/ml of bovine serum albumin (Sigma Chemical Co.) was incubated under sterile conditions with 0.5 M glucose, 1.5 mM PMSF, 0.5 mM EDTA and antibiotics for 6 weeks in phosphate-buffered saline (pH7.4). Non-glycated BSA (BSA) was incubated under same conditions except for the absence of glucose as a negative control. The incorporated glucose was removed by dialysis against PBS using dialysis tube C-75 (Viskase Companies Inc., Darien, IL). After dialysis, BSA and AGE-BSA (AGE) were concentrated by using a filtration device (VIVASPIN 500, 10,000 MWCO PES, Sartorius, Germany). Protein concentrations were determined with a BCA protein assay kit (Sigma Chemical Co.).

### Cell proliferation assay

Cell proliferation activity was measured using a colorimetric Cell Count Reagent SF kit (Nacalai Tesque) according to manufacturer's instruction. Cells were plated in 96-well plates at a density of 3,000 cells/well (ATDC5) or 10,000 cells/well (MC3T3-E1). Cells were treated with BSA or AGE-BSA for 2 days. After cells were incubated with WST-8 for 2 hours, proliferative activities were measured on a microplate reader at 450 nm (model680, Bio-Rad, Tokyo, Japan). There was no difference in the number of dead cells between the cell lines determined by a trypan blue exclusion assay.

### Cell cycle analysis

Each gene transferred cells (1,000,000 cells) in 145 mm dish were analyzed using Millipore Cell Cycle Detection kit (EMD Millipore Corp. Hayward, CA). Cells were incubated for 180 min with fixer, then washed by phosphate buffered saline. Cell cycles of each cells were detected in MUSE cell analyzer (EMD Millipore Corp.).

### Plasmids

Mouse RAGE, dominant negative (DN)-RAGE cDNA were generated by PCR using following oligonucleotides; RAGE cDNA-F 5′-gcGAATTCatgccagcggggacagcagc-3′, RAGE cDNA-R 5′-gcGAATTCttacggtcccccggcaccat-3′ (NM_007425, 1-402aa), for *RAGE*, and RAGE-F plus DN-RAGE cDNA-R 5′-gcGAATTCtcatcgccacaggatagccccga-3′ for DN-*RAGE* (1-362aa), Flag-Cdx1-F (BamHI for pMXs-neo or EcoRI for pUC-CMV-IG) 5′- gcGGATCC(or GAATTC)atggactacaaagacgatgacgacaagatgtacgtgggctatgtgct -3′, Cdx1-R 5′- gcGAATTCctagggtagaaactcctcct-3′ for Cdx1 in a PCR with mouse cDNA pools using KOD plus DNA polymerase (TOYOBO, Osaka, Japan). Similarly, a series of Rho family GTPases also amplified by PCR using primer pair: pRK5-myc-F 5′-gcAGATCTccaccatggaacagaaactcatctc-3′ and pRK5-myc-R 5′-caagcttctgcagGAATTC-3′, and constitutively active or dominant negative form of RhoA, Rac or Cdc42 in pRK5 as a template. Myc-tagged *L63RhoA, L61Rac*, and *L61Cdc42* cDNAs were gifts from Dr. Alan Hall (London University, London, UK). cDNAs were subcloned into pMXs-neo retroviral vector at EcoRI site for *RAGE* or DN-*RAGE*, at BamHI-EcoRI site for Flag-*Cdx1*, BamHI/BglII-EcoRI site for Myc-Rho family GTPases clones. pMXs-IG-neo was generated by subcloning of EcoRI-NotI fragment of IRES-EGFP from pIRES2-EGFP (Clontech Laboratories Inc., Otsu, Japan) and used as negative control. For adenovirus generation, pAC-CMV were modified IRES-EGFP (pAC-CMV-IG) and used as negative control. And then, *RAGE*, DN-*RAGE* or Flag-tagged *Cdx*1 fragments were subcloned into EcoRI site of pAC-CMV-IG. Each adenovirus generating vectors were co-transfected into 293 cells with pJM17, and generated viruses were amplified and monitored through GFP signals.

### Quantitative Real-time PCR

Total RNA was extracted using Sepazol (Nacalai Tesque). One µg of total RNA was reverse-transcribed by ReverTra Ace cDNA synthesis kit (TOYOBO). For quantitative real-time PCR, 5 µl of 2 x KAPA master mix (Nippon Genetics Co. ltd., Tokyo, Japan) was mixed with cDNA, 0.5 µM primer pair. Triplicate of cDNA's were amplified on Piko Real PCR system (Thermo Fisher Scientific Inc., Yokohama, Japan). The experiments were performed at five different cDNA pool dilutions. PCR products were normalized against GAPDH, and measurements between samples were compared by cycling threshold (Ct). Primer sequences used are the following: GAPDH-F 5′- TGCACCACCAACTGCTTAG-3′, GAPDH-R 5′-GGATGCAGGGATGATGTTC-3′, endogenous RAGE-F 5′-CCTGGAGCCTGGGAAGGAAG-3′, 5′- RAGE-F 5′-AGAACATCACAGCCCGGATT-3′, RAGE-R 5′-TTCCTGTGTTCAGTTTCCAT -3′, Runx2-F 5′-CTTCGTCAGCATCCTATCAGTTC-3′, Runx2-R 5′-TCAGCGTCAACACCATCATTC-3′, Opn-F 5′-GCAGAATCTCCTTGCGCCA-3′, Opn-R 5′-CGAGTCCACAGAATCCTCGC-3′, Ocn-F 5′-CGCTCTGTCTCTCTCTGACCTC-3′, Ocn-R 5′-GACTGAGGCTCCAAGGTAGC-3′, Ihh-F 5′-TTCAAGGACGAGGAGAACACG-3′, Ihh-R 5′-TTCAGACGGTCCTTGCAGC-3′, PTHrP-F 5′- GGCGTTCGGTGGAGGGGCTT -3′, PTHrP-R 5′- CAGATGGTGGAGGAAGAAAC -3′, PTHrPR-F 5′-GCTGCTCAAGGAAGTTCTGC-3′, PTHrPR-R 5′-TCTCTTTAGACTCGGGGTAG-3′, col2a1-F 5′-TCGCCATAGCTGAAGTGGAAG-3′, col2a1-R 5′-ACTGTCCCTCGGAAAAACTGG-3′, col10a1-F 5′-TTCAGGGAGTGCAATCATGGAG-3′, col10a1-R 5′-GCAATTGGAGCCATACCTGGTC-3′, Sox9-F 5′- TGGACATCGGTGAACTGAGC -3′, Sox9-R 5′- AGTGTAGGTGACCTGGCCGT -3′, MMP-13-F 5′- TCACCTGATTCTTGCGTGCT -3′, MMP13-R 5′- CTGTGGGTTATTATCAATCTTGTTTCTT -3′, VEGF-F 5′-GGACCCTGGCTTTACTGCTG-3′, VEGF-R 5′-TCGCTGGTAGACGTCCATGA-3′, ALP-F 5′-GTGGAAGGAGGCAGGATTGAC-3′, ALP-R 5′-GCTTCATGCAGAGCCTGCTT-3′, Cdx1-F 5′-CTAGGACAAGTAGCTTGCCCTCTT-3′, Cdx1-R 5′-TCCAACAGGCTCACCACACA-3′, Cdx2-F 5′-CGATACATCACCATCAGGAGG-3′, Cdx2-R 5′-TGGCTCTGCGGTTCTGAAAC-3′, Cdx4-F 5′-GAGGAAGTCAGAGCTGGCAG-3′, Cdx-4-R 5′-GGCTCTGCGATTCTGAAACC-3′, RANKL-F 5′-CAAGCTCCGAGCTGGTGAAG-3′, RANKL-R 5′-CCTGAACTTTGAAAGCCCCA-3′, OPG-F 5′-AAGAGCAAACCTTCCAGCTGC-3′, OPG-R 5′-CACGCTGCTTTCACAGAGGTC-3′. The non-regulated housekeeping gene GAPDH served as an internal control and was used to normalize for differences in input RNA. All measurements were performed in quadruplicate.

### Reporter assay

Reporter assays were performed by transient transfection of 0.1 µg of p1300-luc, human RAGE promoter construct (−1689/+43) [33], Ihh-luc [34], Col10a1 p3000-luc [35], and 0.002 µg of pRL-CMV (Promega, Madison, WI) using Dual Luciferase Reporter Assay System (Promega) as previously described [29]. Reporter assays using NF-kB-luc (Agilent tech., Santa Clara, CA) were performed using ONE-Glo Luciferase Assay System (Promega). Two genomic clones were prepared by PCR-based method. Mouse RANKL promoter was subcloned into SacI-HindIII site of pGL4.10 basic vector (Promega) using following primer pair: RANKL pro-F 5′-CGAGCTCAGAATGAGGTGGTGGTCTTGCAG-3′, RANKL pro-R 5′- CCAAGCTTGGCGCGGCGCCCGGAGTTCG-3′. Mouse OPG promoter was subcloned into MluI-XhoI site of pGL3 basic vector (Promega) using following primer pair: OPG pro-F 5′-gcACGCGTacatccagagccaagagctg-3′, OPG-pro-R 5′-gcCTCGAGgcgcggaggcgtgggacaag-3′. Luciferase activity was measured using a model TD20/20n luminometer (Turner BioSystems, Sunnyvale, CA).

### Micromass culture

For micromass culture, limb buds from E12.5 embryos were isolated and were digested in 0.1% trypsin/0.1% collagenase for 30 min at 37°C as described previously [32]. Briefly, cells were suspended by pipetting and reaction was stopped by Dulbecco's Modified Eagle Medium (DMEM) (WAKO Pure Chemical Industries, Ltd., Osaka, Japan) containing 10% fetal bovine serum (FBS). Cells were resuspended in 20 µl drops at 2×10^7^ cells/ml, plated to 12-well plates. After 1 hour to allow the attachment of cells, 2 ml of DMEM containing 10% FCS were overlaid. After 24-h, chemical treatment or virus infection were started. And then, alucian blue staining was performed to detect the production of cartilaginous matrix as described previously [32]. Medium was changed every day. For alucian blue staining, the wells were rinsed twice with PBS and fixed with methanol for 5-min. They were then stained overnight with 0.1% alucian blue in 20% acetic acid/80% ethanol. Wells were rinsed three times with distilled water and dye was extracted with 6 M guanidine-HCl. Absorbance of extracted dye was measured at 620 nm.

### Stable transfected cells

For establishment of NF-kB-luc stable transfected cells, neomycin resistant gene cassette from pEGFP-N1 (Clontech Laboratories Inc.) was subcloned into NdeI site by PCR using following primers; EcoRI-NdeI-neo-F 5′-gcGAATTCATATGgtgtggaaagtccccag-3′, EcoRI-NdeI-BamHI-neo-R 5′-gcGAATTCATATGGATCCtttattctgtct-3′. For establishment of Flag-Cdx1 stable transfected cells, pRC/CMV-Flag Cdx1 (gifted from Dr. John P. Lynch, University of Pennsylvania [36]) and pEGFP-N1 were co-transfected to the cells. Stable transfected cells were selected by G418 and cell colonies were isolated independently.

### Chromatin immunoprecipitation

Chromatin immunoprecipitation was performed as described previously [34]. Briefly, GFP control cells or Flag-tagged *Cdx1* cells were fixed by paraformaldehyde and fragmented genome were prepared by sonication (Bronson sonifier model 150, setting 5, 5sec-25-times). After immunoprecipitation using anti-Flag M2 beads (Sigma Chemical Co.), DNA was purified from supernatants and immunoprecipitates. Real-time PCR was performed using the following primers specific to the promoter region of mouse Ihh gene: Ihh-Cdx-F, 5′-TGGATTGGGATCCGTTTGTT-3′; Ihh-Cdx-R, 5′-AAGCAAACCCGAAAGCTGGA-3′. From the experiment using anti-HA antibody (Santa Cruz Biotechnology, Inc., Y-11), we confirmed the specificity of pull-down by M2 beads.

### Statistical analysis

Data are expressed as mean ±S.E.M. Significance was tested using a Student's t-test or, where multiple comparisons were required, Tukey-test or Williams-test. A P-value of less than 0.05 was considered to be significant.

## Results

### The expression of RAGE in embryo skeleton

**Figure 1:**
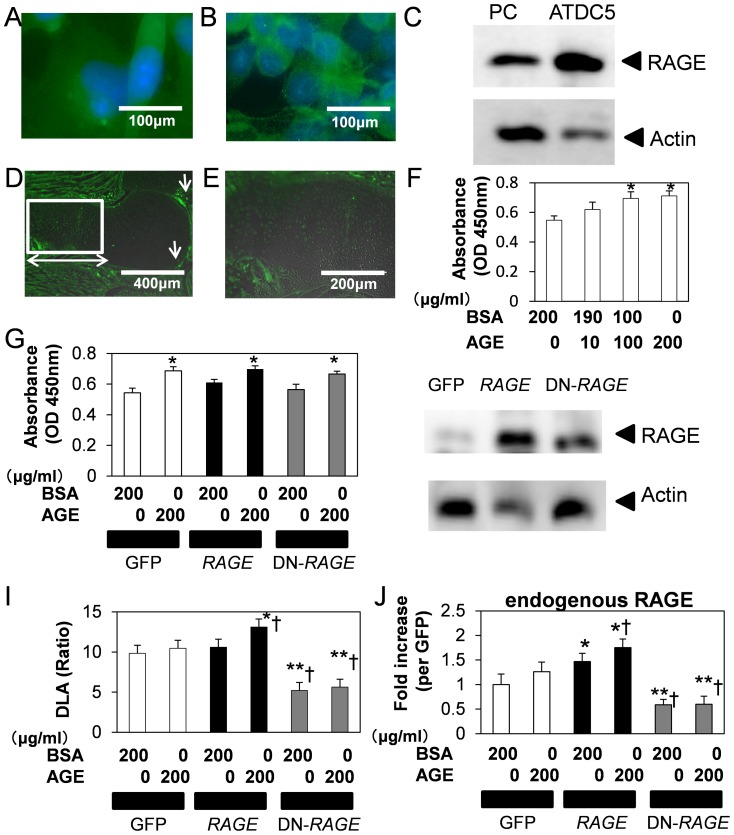
RAGE expressed in chondrocytes both in *in vitro* and *in vivo*. RAGE expression in primary chondrocytes (A), and in chondrogenic ATDC5 (B). Staining procedures were described in “Materials and Methods”. RAGE antibody positive reactions (Green) were observed in membranous regions. Cells were counter stained by DAPI (Blue). Refer to figure S5. C, RAGE expressions in cultured cells. Primary chondrocytes (PC) and ATDC5 cells were cultured up to confluent and then cells were lyzed. Immunoblot analysis was performed as described in “Materials and Methods”. (D, E) RAGE expression in cartilage. Immunostaining of hindlimb isolated from mouse embryos at 15.5 days post-gestation at low (D) and high (E) magnification (indicated as square in D). Refer to figure S5. RAGE expressed from prehypertrophic to hypertrophic zone of femur (double-headed arrow). The arrow shows area of non-specific reaction. F, AGEs (AGE) stimulated chondrogenic ATDC5 cells proliferation. ATDC5 cells were treated with indicated concentrations of BSA or AGE-BSA (AGE) for 48-h, and cell proliferation was evaluated by WST-8 incorporation calorimetrically. Values are expressed as the mean ±SEM of 8–16 wells. *P<0.05 vs BSA alone. (G, H) RAGE dependency of AGEs-induced ATDC5 cells proliferation. To examine RAGE-dependency, we established stable transfected cells by retrovirus infection. G, Respective gene-transferred cells were treated with indicated concentrations of BSA or AGE and cell proliferation were examined. Increased cell proliferation by AGE compared to BSA control in GFP cells was reproduced in *RAGE*, or DN-*RAGE* cells at similar levels. Values are expressed as the mean ±SEM of 8–16 wells. *P<0.05 vs BSA alone. The expression levels of RAGE in each infectants are shown in H. (I–J) RAGE promoter activity and RAGE expression regulated by RAGE in ATDC5 cells. I, Respective cells were treated with indicated concentrations of BSA or AGE 3-h after transfection. Dual luciferase activities (DLA) were evaluated at 24-h after transfection. AGEs did not promote RAGE promoter activity in GFP cells, and stimulated in *RAGE* cells significantly. Suppressed RAGE promoter activity was observed in DN-*RAGE* cells. Values are expressed as the mean ±SEM of 8 wells. J, Endogenous RAGE expression levels in response to AGE. Cells were treated with indicated concentrations of BSA or AGE. RAGE expression levels were examined by real-time-PCR using primer pair designed to detect endogenous RAGE expression. The value corrected by Gapdh, was expressed as 1 GFP control. Values are expressed as the mean ±SEM of 4 wells. *P<0.05, **P<0.005 vs BSA in GFP cells, †P<0.05 vs AGE in GFP cells. Similar results were obtained from additional three experiments.

AGEs are known as RAGE ligands and exert mitogenic action in vascular smooth muscle cells [33]. We first examined RAGE immunoreactivity in primary chondrocytes and chondrogenic ATDC5 cells. Following antibody reaction before fixation, RAGE was observed around the cell membrane in chondrocytes (Fig.1A, B). RAGE expression levels in primary chondrocytes and ATDC5 cells were similar (Fig.1C). To elucidate physiological expression of RAGE in *in vivo*, we tested RAGE immunoreactivity in embryonic hind limbs (Fig.1D, E). RAGE was mainly expressed from the prehypertrophic to hypertrophic region and perichondorium in E15.5 hind limbs. Reactivity against RAGE antibody was only weakly observed in proliferative chondrocytes, and negligibly in cancellous bone, periosteum and bone collar. These results suggested that RAGE regulates chondrocyte in the prehypertrophic to hypertrophic stages.

### RAGE-independent ATDC5 proliferation

Using prepared BSA or AGE-BSA (AGE), we first examined the effect of AGE on ATDC5 proliferation. AGE significantly promoted ATDC5 proliferation in a concentration-dependent manner (Fig.1F). On the other hand, AGE had no effect on osteoblastic MC3T3-E1 cells (Fig.S1G). In attempt to examine the dependency of RAGE on chondrocyte proliferation, stable ATDC5 transfected cells expressing GFP, *RAGE* or DN-*RAGE* were established using retroviruses (Fig.1G–J). Mitogenic action of AGE was not influenced by overexpression of *RAGE* or DN-*RAGE* compared to GFP control cells (Fig.1G). RAGE expression levels in *RAGE* or DN-*RAGE* cells were clearly elevated (Fig.1H). In addition, basal cell cycle status of these cells was unaffected (Figl.S1A–F). Although slightly, the progression into S phase was inhibited by *RAGE*. These results suggested that AGEs induced ATDC5 proliferation in a RAGE-independent manner. A previous report demonstrated that AGEs activate human RAGE promoter [33]. Therefore using stable transfected cells of *RAGE* or DN-*RAGE*, we examined the effect of AGE on human RAGE promoter activities (Fig.1I). Elevated RAGE promoter activity by AGE was tended to facilitate by *RAGE*. Basal and AGE-induced RAGE promoter activities were inhibited by DN-*RAGE* significantly. Using a specific primer pair to detect endogenous RAGE, real-time PCR analysis demonstrated that RAGE by *RAGE* overexpression with or without AGE was significantly upregulated, and down regulated by DN-*RAGE* (Fig.1J). These results suggested that extracellular AGE stimulation influences chondrocyte function, DN-*RAGE* functions as dominant negative form and that AGE regulated RAGE expression.

### The role of RAGE on chondrocytes differentiation

**Figure 2:**
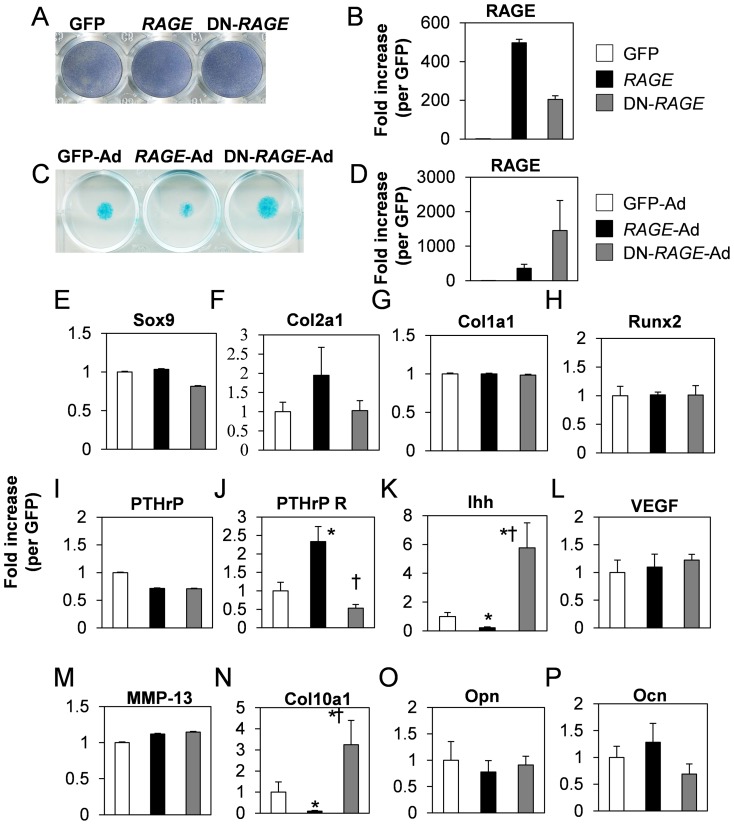
Cartilaginous matrix production and gene regulation by RAGE. (A, B) RAGE did not modulate ALP activities. A, Established stable transfected cells were stained by ALP in ATDC5 cells. ALP activity levels were not influenced by *RAGE* or DN-*RAGE*. Transgene levels are shown in B. (C, D) RAGE inhibited cartilaginous matrix production. Staining procedures were mentioned in “Materials and Methods”. In micromass culture, cells were infected with adenoviruses expressing GFP (GFP-Ad), *RAGE* (*RAGE*-Ad), or DN-*RAGE* (DN-*RAGE*-Ad) at approximately 50MOI. Transgene levels are shown in D. The value are normalized against Gapdh. (E–P) ATDC5 cells were infected by GFP-Ad, *RAGE*-Ad, or DN-*RAGE*-Ad. At day 2 post infection, mRNA expression levels were monitored by real time-PCR analysis using respective specific primer pairs described in “Materials and Methods”. Values are expressed as the mean ±SEM of 4 wells. *P<0.05 vs GFP-Ad, †P<0.001 vs *RAGE*-Ad. Similar results were obtained from additional three experiments.

To monitor differentiation state of stable transfected cells of GFP, *RAGE* or DN-*RAGE*, we performed ALP staining (Fig.2A). ALP transcripts in each stable transfected cells were not influenced (data not shown). Next, we performed micromass culture to monitor cartilaginous matrix production. After isolation of limb buds from E12.5 mouse embryos, cells were infected with GFP-Ad, *RAGE*-Ad, or DN-*RAGE*-Ad. Two days later after infection, cells were stained by alucian blue (Fig.2C). The results showed that *RAGE*-Ad inhibited cartilaginous matrix production and DN-*RAGE*-Ad augmented production compared to GFP-Ad control. To investigate RAGE action on gene regulation, mRNA expression levels were analyzed by real-time PCR. In ATDC5 cells, Sox9, Col1a1, Col2a1, Runx2, PTHrP, VEGF, MMP-13, osteopontin and osteocalcin expressions were not changed by *RAGE*-Ad or DN-*RAGE*-Ad, while Ihh, col10a1 were downregulatd by *RAGE*-Ad and upregulated by DN-*RAGE*-Ad (Fig.2E–I, K–P). Conversely, PTHrP receptor (PTHrP R) was significantly upregulated by *RAGE*-Ad (Fig.2J). This data strongly suggested that RAGE negatively regulated chondrocyte differentiation at the prehypertrophic stage.

### NF-kB-independent and Rho family GTPases-dependent mechanisms

**Figure 3:**
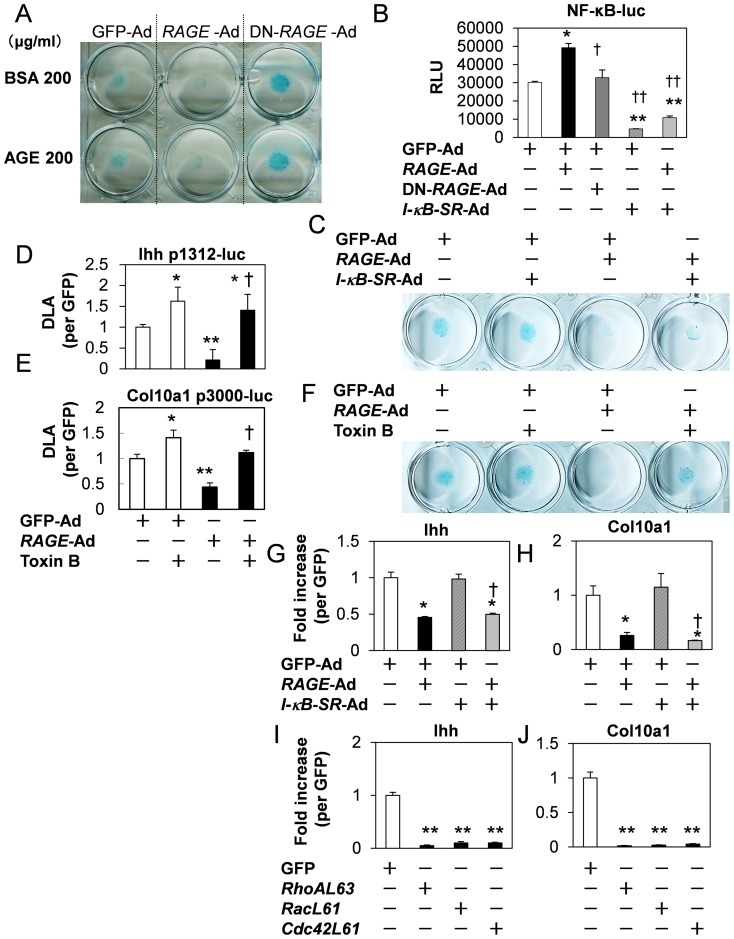
NF-κB-independent and Rho family GTPases-dependent mechanisms. A, RAGE independency of AGEs-induced cartilaginous matrix production. Cells were infected with indicated adenoviruses at approximately 50MOI, then treated with BSA and AGE. 2 days after, cells were stained by alucian blue. B, The inhibitory action to NF-κB activities by *I-κB-SR*-Ad. Respective adenoviruses were infected at approximately 50 MOI to established stable transfected cells of NF-κB-luc in ATDC5 cells. 24-h after infection, cells were lyzed and analyzed NF-κB activities. Relative luciferase units (RLU) were shown. *P<0.05 vs GFP-Ad, †P<0.05, ††P<0.001 vs *RAGE*-Ad. n = 8. C, *I-κB-SR*-Ad failed to rescue *RAGE*-dependent cartilaginous matrix production in micromass culture. 2 days after infection at approximately 25 MOI, cells were stained by alucian blue. (D,E) Toxin B restored Ihh and Col10a1 promoter activities downregulated by RAGE. ATDC5 cells were transfected with Ihh p1312-luc (D) or Col10a1-luc (E) with pRL-CMV, and respective adenoviruses were infected at approximately 50 MOI at same time. 3-h after gene transfer, cells were treated with 100 pg/ml Toxin B. Values are expressed as the mean ±SEM of 4–8 wells. *P<0.05, **P<0.001 vs GFP-Ad without Toxin B, †P<0.001 vs *RAGE*-Ad without Toxin B. F, Toxin B restored the inhibition of *RAGE*-dependent cartilaginous matrix production in micromass culture. Respective adenoviruses were infected at approximately 50 MOI. 3-h after infection, cells were treated with 100 pg/ml Toxin B. (G, H) *I-κB-SR*-Ad did not influence Ihh (G) and Col10a1 (H) expressions in ATDC5 cells. 2 days after infection at approximately 25 MOI, total RNAs were prepared. *P<0.05 vs GFP-Ad, †P<0.05 vs *RAGE*-Ad. n = 4. (I, J) Rho GTP ases activation causes the reduction of Ihh and Col10a1 in ATDC5 cells. Expression levels of Ihh (J) and Col10a1 (K) transcripts were monitored by real-time PCR analysis. After confluency, total RNAs were prepared from respective stable transfected cells. Values are expressed as the mean ±SEM of 4 wells. **P<0.001 vs GFP. Similar results were obtained from additional three experiments.

To examine dependency on AGE of RAGE effects on cartilaginous matrix production, we tested the effect of AGE in GFP-, *RAGE-* and DN-*RAGE*-infected cells using adenoviruses (Fig.3A). Results showed that AGE did not influence cartilaginous matrix production in comparison to BSA treatment in all infected cells. On the other hand, one candidate ligand for RAGE, HMGB1 clearly inhibited them in a concentration- and *RAGE*-dependent manner (Fig.S3A, B). To clarify the involvement of NF-κB signal that is known to be promoted by RAGE, we assessed NF-κB activity. For this, we established NF-κB-luc stable transfected cells of ATDC5. NF-κB activity was stimulated by *RAGE*-Ad but not by DN-*RAGE*-Ad (Fig.3B). HMGB1 stimulated NF-κB activity in GFP-Ad transfected cells, but not in DN-*RAGE*-Ad transfected cells (Fig.S3C). I-κB inhibitor, *I-κB*-*SR*-Ad effectively inhibited basal and *RAGE*-induced NF-κB activities. *I-κB*-*SR*-Ad did not affect cartilaginous matrix production in both GFP-Ad and *RAGE*-Ad (Fig.3C). Additionally, the inhibitory effects on Ihh and col10a1 mRNA expressions by *RAGE*-Ad were not influenced by *I-κB*-*SR*-Ad (Fig.3G, H). These results suggested that RAGE inhibited chondrocyte differentiation in a NF-κB-independent manner.

Next, we investigated downstream RAGE signaling. Recent report on chondrocyte maturation signals suggests that there is significant cross-talk among the pathways and that the overall effects on chondrocyte function is dependent on the balance in activity of multiple signaling proteins [37]. We first tested Toxin B, a Rho family GTPases inhibitor action. As shown in Fig.3F, Toxin B solely did not influence cartilaginous matrix production and restored *RAGE*-Ad-induced inhibitory action. In reporter analysis, Toxin B restored *RAGE*-Ad-induced inhibitory effects on Ihh and Col10a1 promoter activities without influencing NF-κB activity (Fig.3 D, E, and data not shown). We next analyzed effect of the activation state of Rho family GTPases. We established stable transfected cells of constitutively active forms of Rho family GTPases, *L63RhoA, L61Rac* or *L61Cdc42* in ATDC5 by infection using retroviruses. Real-time PCR analysis showed decreased Ihh and Col10a1 mRNA expressions in *L63RhoA, L61Rac*, or *L61Cdc42* (Fig.3I, J). These results suggested that RAGE inhibited chondrocyte differentiation in a Rho family GTPases-dependent manner.

### Inhibitory mechanism of chondrocyte differentiation by RAGE

**Figure 4:**
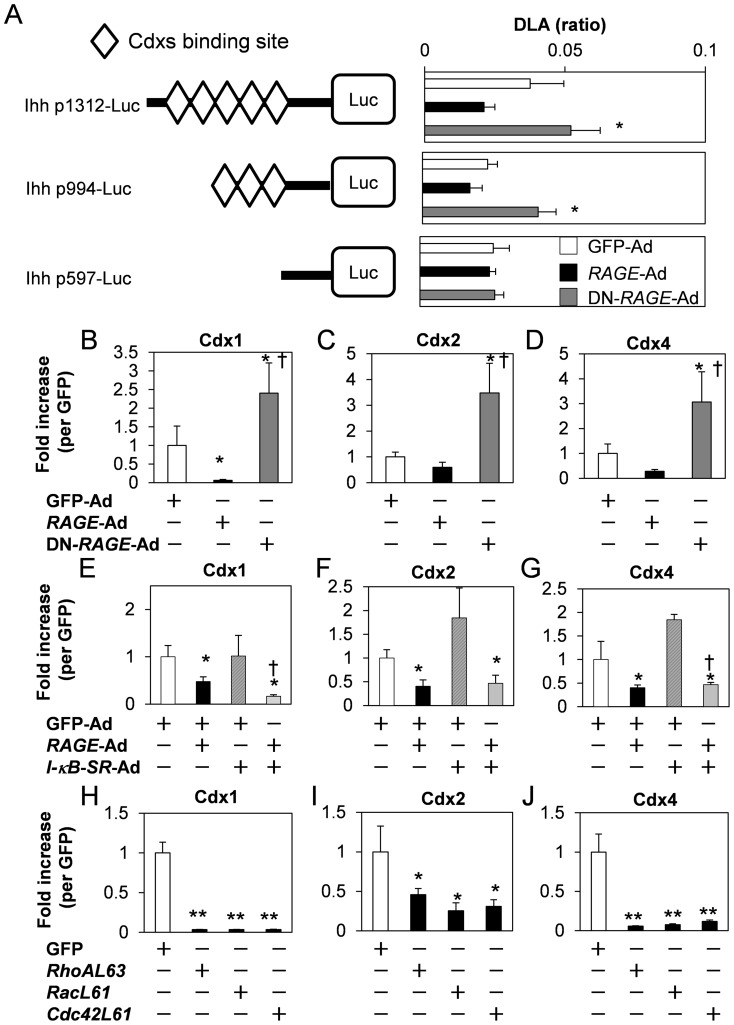
Relationship between Cdx and Ihh, and downregulation by RAGE. A, Ihh promoter activities regulated by RAGE. Cells were transfected with indicated Ihh-luc constructs (left panel). 3-h after transfection, cells were infected with GFP-Ad, *RAGE*-Ad, or DN-*RAGE*-Ad at approximately 50MOI. Ihh promoter activities reduced by *RAGE*-Ad and increased by DN-*RAGE*-Ad were monitored in p1312-luc and p994-luc, while in p597-luc, *RAGE*-Ad and DN-*RAGE*-Ad did not influence to promoter activity (right panel). Values are expressed as the mean ±SEM of 4–8 wells. *P<0.05 vs GFP-Ad. (B–D) Cdxs regulation by RAGE. cDNA pool was used same as in Fig.2. *P<0.05 vs GFP-Ad, †P<0.05 vs *RAGE*-Ad. Values are expressed as the mean ±SEM of 4 wells. (E–G) NF-κB-independent downregulation of Cdxs by RAGE. cDNA pool was used same as in Fig.3G and H. *P<0.05 vs GFP-Ad,†P<0.05 vs *RAGE*-Ad. Values are expressed as the mean ±SEM of 4 wells. (H–J) Rho GTP ases activation cause the reduction of Cdxs. cDNA pool was used same as in Fig.3I and J. **P<0.001 vs GFP. Values are expressed as the mean ±SEM of 4 wells. Similar results were obtained from additional three experiments.

To explore the precise mechanism of chondrocyte differentiation regulated by RAGE, we analyzed inhibition of Ihh expression by *RAGE* using Ihh promoter deletional constructs [34]. *RAGE*-induced inhibitory action and DN-*RAGE*–induced stimulatory action were maintained in p1312-luc and p994-luc, while these actions were disrupted in p597-luc (Fig.4A). To determine *RAGE*-dependent responsive DNA elements within region from −994 to −597 in Ihh promoter, we performed in silico anaysis using TF Search program (http://www.cbrc.jp/research/db/TFSEARCH.html) (Fig.S2). From transcription factor binding sites within 397 bp of Ihh promoter sequence, Cdxs were identified as candidate factors modulated by RAGE. Real-time PCR analysis demonstrated that Cdxs expression was inhibited by *RAGE*-Ad and stimulated by DN-*RAGE*-Ad (Fig.4B–D). To examine the dependency on the NF-κB pathway, we tested effect of *I-κB-SR*-Ad on *RAGE*-induced Cdxs downreduction (Fig.4E–G). *I-κB*-*SR*-Ad did not influence Cdxs expression with or without *RAGE*-Ad. In addition, *L63RhoA, L61Rac* or *L61Cdc42* showed decreased Cdxs expressions in comparison to GFP control (Fig.4H–J).

### Characterization of Cdxs as a target molecule of RAGE signaling

**Figure 5:**
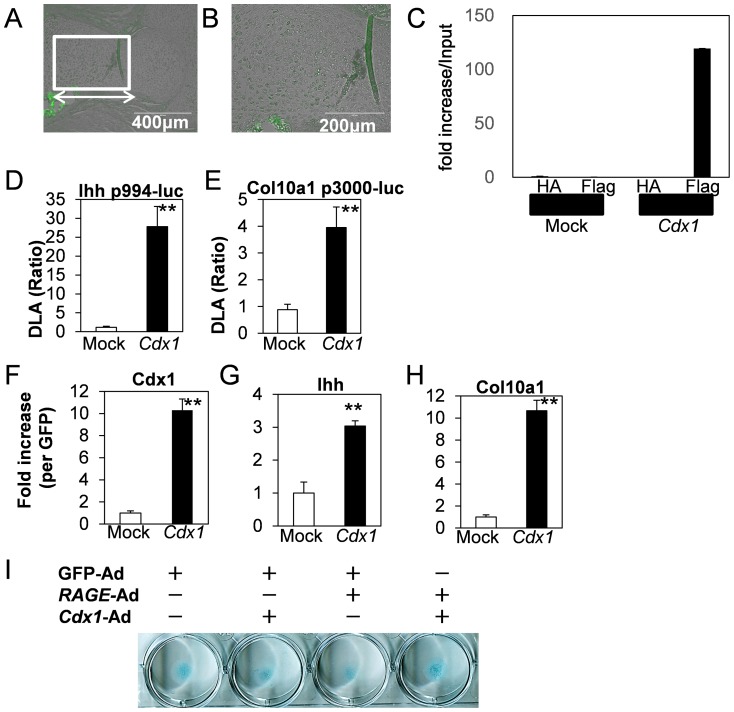
Cdx1 regulated chondrocytes differentiation. (A, B) Cdx localization in fetal skeleton. Immunohistochemical analysis was performed using serial section as shown in Fig.1D, E, Cdx1 expressed from prehypertrophic to hypertrophic zone of femur (double-headed arrow) and B showed magnified image indicated as square in A. Refer to figure S6. C, The interaction between Cdx1 and Ihh promoter by ChIP analysis. Fragmented chromatins of MOCK or *Cdx1* cells in ATDC5 were immunoprecipitated by FLAG M2 beads. Amplification input DNA was constant. Immunoprecipitated DNA-derived amplification by Flag-M2 beads was detected only in *Cdx1* cells. (D, E) Ihh and Col10a1 promoter regulation by *Cdx1*. ATDC5 cells were transiently transfected with indicated promoter constructs with pRL-CMV. DLA was performed 24-h after transfection. Elevated promoter activities of Ihh (D) and Col10a1 (E) in *Cdx1* cells were observed compared to MOCK control. **P<0.001 vs MOCK. Values are expressed as the mean ±SEM of 4 wells. (F-H) Ihh and Col10a1 regulated by *Cdx1*. mRNA expression levels were monitored by real-time PCR analysis. **P<0.001 vs MOCK. Values are expressed as the mean ±SEM of 4 wells. I, Cdx1 restored cartilaginous matrix production inhibited by RAGE. Respective adenoviruses were infected at approximately 25 MOI at same time. Similar results were obtained from additional three experiments.

We examined Cdx1 expression pattern in *in vivo* (Fig.5A, B). Cdx1 immunoreactivity was abundant in prehypertrophic chondrocytes and poor in proliferative and hypertrophic chondrocytes and the perichondorium. On the other hand, no expression was observed in cancellous bone, periosteum and bone collar at E15.5. Next, to determine the direct interaction of Cdx1 and Ihh promoter, we performed chromatin immunoprecipitation (ChIP) assay. For this, we established stable transfected cells of MOCK or Flag-tagged *Cdx1* in ATDC5 cells. Approximately, a ten-fold increase of Cdx1 expression was observed in Flag-tagged *Cdx1* cells (Fig.5F). ChIP analysis showed direct interaction between Cdx1 and Ihh promoter (Fig.5C). Transient overexpression studies demonstrated that *Cdx1* stimulated Ihh p994-luc and col10a1 p3000-luc promoter activities (Fig.5D, E). In stable transfected cells, *Cdx1* upregulated Ihh and Col10a1 expressions compared to MOCK control (Fig.5G, H). Additionally, *Cdx1*-Ad restored *RAGE*-Ad-induced inhibition of cartilaginous matrix production (Fig.5I).

**Figure 6:**
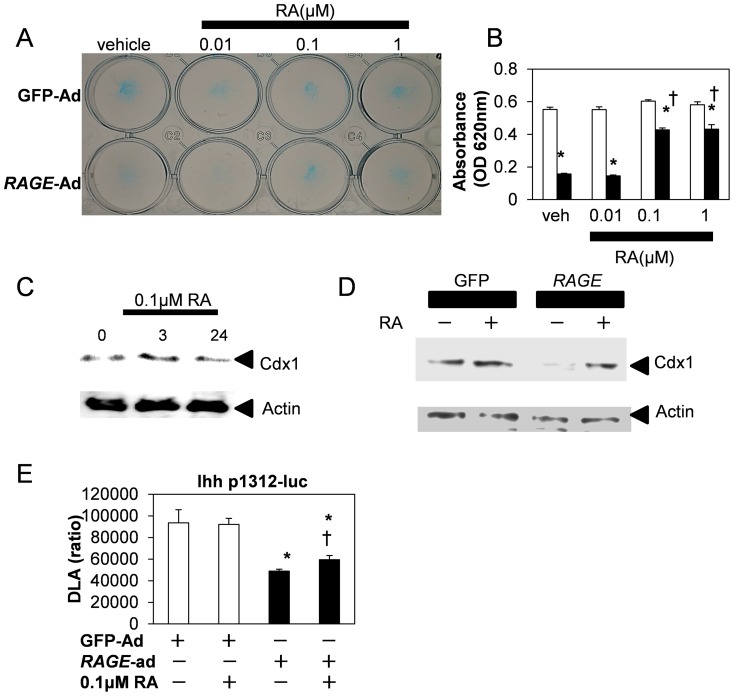
RAGE-dependent inhibition of chondrocytes differentiation rescued by RA. A, RAGE-induced inhibition of cartilaginous matrix production was rescued by RA. Indicated concentrations of RA were added 3 hours after infection of GFP-Ad, or *RAGE*-Ad at approximately 50 MOI. 2-days later, alucian blue staining was performed and quantitative data were obtained and summarized in B. *P<0.001 vs GFP, †P<0.001 vs *RAGE*-Ad without RA. Values are expressed as the mean ±SEM of 4 wells. C, Transient induction of Cdx1 by RA. Immunoblot analysis showed that 0.1 µM RA maximally stimulated Cdx1 at 3 h after treatment. D, Comparison of Cdx1 induction in GFP and *RAGE* cells. Using established stable transfected cells, Cdx1 induction by RA treatment was examined by immunoblot analysis. After confluency, GFP or *RAGE* cells were treated for 3 h in the absence or the presence of 0.1 µM RA. E, Downregulated Ihh promoter activity by RAGE was partially recovered by RA. 0.1 µM RA was added at 3-h after infection of indicated adenoviruses. *P<0.05 vs GFP-Ad,†P<0.05 vs *RAGE*-Ad. n = 4.

Previous reports showed that Cdx1 is a direct target gene of retinoic acid (RA) [38]. Therefore we examined whether or not RA would affect *RAGE* inhibitory action. RA rescued *RAGE*-induced inhibition of cartilaginous matrix production in a concentration dependent fashion (Fig.6A, B). 0.1 µM RA exerted protective action on cartilaginous matrix production inhibited by *RAGE*-Ad. RA also induced Cdx1 expression transiently (Fig.6C). We examined the difference of RA response using GFP and *RAGE* stable transfected cells (Fig.6D). Immunoblot analysis showed that Cdx1 expression was reduced in *RAGE* cells compared to GFP cells without RA treatment, and Cdx1 induction by 0.1 µM RA was clearly observed in *RAGE* cells. RA itself did not influence Ihh promoter activity (Fig.6E). Reduced Ihh promoter activity caused by *RAGE*-Ad was partially restored after 0.1 µM RA treatment.

## Discussion

Damage to the cartilage is a major problem, especially in joint disease. AGEs are one of the candidates that act as adverse mediators in joint diseases [22], [23]. RAGE has been shown to be an initiator of inflammatory response in immune responsive cells such as macrophages [39]. Although RAGE knockout mice show skeletal abnormalities after birth [15], [19], the biological importance of RAGE expression in chondrocytes remains to be clarified. Hence in this work, we examined the precise mechanisms, 1; regulatory mechanism of chondrocytes functions by RAGE, 2; involvement of most conceivable inflammatory NF-κB or Rho family GTPases signals in chondrocytes. We showed for the first time that RAGE activation in chondrocytes functions as a mediator for suppression of chondrocyte differentiation via NF-κB-independent and Rho family GTPases-dependent mechanisms.

### RAGE as a negative regulator of cell differentiation

Previous reports showed that AGEs stimulate human vascular endothelial cell proliferation [33]. As there was a possibility that AGEs action might be cell type specific, we examined AGEs action on chondrocyte proliferation (Fig.1F). Overexpression studies demonstrated that RAGE did not function as mitogen in chondrocytes (Fig.1G), suggesting that the mitogenic action of AGEs is mediated via receptor(s) other than RAGE. Immunohistochemical analysis showed that RAGE expression was mainly in prehypertrophic stage chondrocytes and the perichondorium at E15.5 (Fig.1D, E). Furthermore, AGEs did not influence to MC3T3-E1 proliferation (Fig.S1G). These results suggested that during fetal development RAGE was functional in chondrocytes but not in osteoblasts. On the other hand, knockout mice of Hmgb1, an endogenous candidate ligand for RAGE, had abnormal skeletal phenotype during fetal development [26], [27]. These observations suggest that Hmgb1 probably controls skeletal formation in a RAGE-independent manner during fetal development. As our results indicated that AGE slightly influences proliferation but not cartilaginous matrix production (Fig.1F, Fig.3A), and AGE influenced RAGE expression (Fig.1I, J), exogenous HMGB1 clearly inhibited cartilaginous matrix production (Fig.S3), suggesting that AGE somewhat influences chondrocytes function, HMGB1 may function as key factor in some pathological or physiological conditions. Taking all this into consideration, we hypothesize that AGE accumulation is as a result of physical flexibility and stiffness as opposed to receptor activation by bioactive molecules. Further investigation for the role of AGE-RAGE signaling axis will be required.

We examined RAGE function in the absence of ligand stimulation since *RAGE* overexpression solely evoked significant receptor activation as shown in Fig.3B. This could be attributed to stimulation by FCS-derived factors. The effect of AGE as a RAGE ligand was limited in our systems as described above. In attempt to unravel how RAGE regulates chondrocytes, we designed overexpression experiments studies using *RAGE* and DN-*RAGE*. Pathologically, RAGE is expressed in articular chondrocytes and is thought to mediate AGE-induced osteoarthritis [22]. In human articular cartilage, an increase in AGE levels negatively affects proteoglycan synthesis thereby reducing cellular turnover and repair capacity hence contributing to tissue degradation [23]. We assumed from the pathologic high expression of RAGE after birth that RAGE does play a functional role in chondrocytes. *RAGE* clearly inhibited cartilaginous matrix production (Fig.2C), influenced expression of two prehypertrophic chondrocytes differentiation markers, Ihh and PTHrP receptors and the hypertrophic chondrocyte differentiation marker Col10a1 (Fig.2J, K, N). These results demonstrated that even without AGE stimulation, overexpression of RAGE activation was sufficient to inhibit chondrocytes differentiation.

We also demonstrated that Cdxs identified from the Ihh promoter by in silico analysis, upregulated Ihh and Col10a1 expressions (Fig.4A–D, Fig.5G, H). The Cdx (caudal) gene family of homeodomain transcription factors, Cdx1, Cdx2, and Cdx4, are thought to act as modulators of vertebral axial patterning [40]. Cdx1 and Cdx2 are expressed in the primitive streak region at E7.5, followed by Cdx4 expression at E8.5 [41]. Spatio-temporally regulated Cdx expression has been proposed to be indicative of a functional Cdx gradient that regulates spatial expression of target genes along the major body axis [42], [43]. Cdxs are regulated by Homeobox genes, which modulate spatio-temporal gene expression to form precise body axis [44]–[47]. It is considerable that Cdxs are downregulated by *RAGE*, subsequently suppressing the expression of Ihh and Col10a1. Indeed, Cdxs gradient is important in the formation of the body axis [41], and RAGE disturbed Cdxs expression levels in cultured cells at least. Metabolic cartilaginous diseases cause deformation in joints. Excessive RAGE accumulation in joint diseases has been reported [23], [24]. This suggests that pathological RAGE activation probably causes disturbance of Cdxs, in turn leading to skeletal deformation. Based on these observations, we investigated the effect of *Cdx1* overexpression on *RAGE*-induced cartilaginous matrix production and on gene regulation (Fig.4B–D). Cdx1 was expressed from the prehypertrophic to hypertrophic region of chondrocytes an expression pattern similar to that of RAGE (Fig.1D, E, Fig.5A, B). Additionally, *RAGE* overexpression downregulated Cdx1 (Fig.4B–D). DN-*RAGE* overexpression clearly inverted gene expressions, suggesting that Ihh, Col10a1, and Cdxs were RAGE-sensitive genes. These processes were all NF-κB-independent (Fig.3C, G, H and Fig.4E–G). The downstream RAGE signals include small GTPases Rho family protein functions as mediators [25]. RAGE inhibitory action was restored by the Rho family GTPases inhibitor Toxin B (Fig.3D–F) while constitutively active forms of all Rho GTPases downregulated Cdxs, Ihh and Col10a1 (Fig.3I, J, Fig.4H–J). In addition, Cdx1 clearly facilitated chondrocyte differentiation (Fig.5G–I). Altogether these findings suggests the RAGE-Rho GTPases signaling axis functions as a down regulator for Ihh by modulating Cdxs in prehypertrophic stage chondrocytes.

### Regulation of spatiotemporal Ihh signal by RAGE

Previous studies on osteoclasts regulation by RAGE showed that RAGE functions as a transducer for osteoclast maturation signal [15], [19]. Long bones in skeletal parts by endochondral ossification were elongated in RAGE knockout mice compared to wild type mice after birth. Elongated long bone length observed in RAGE knockout mice is suggestive of the possibility of additional RAGE downstream signals besides the osteoclast regulation pathway. Ihh is an essential factor for cartilaginous longitudinal development and RAGE activation resulted in Cdxs-Ihh downregulation (Fig.2K, Fig.4B–D). On the other hand, the disruption of RANKL, an essential osteoclast stimulator, caused dwarfism by osteopetrosis [48]. The binding of RANKL to its receptor RANK, promotes osteoclast maturation and activation in the NF-κB pathway. While, *RAGE*-Ad stimulated NF-κB in chondrocytes (Fig.3B), *I-κB-SR-*Ad did not influence gene regulation of Ihh and Col10a1 (Fig.3G, H). Additionally, we studied whether RAGE affects the RANKL and OPG expressions or not (Fig.S4A, B). Interestingly, RAGE failed to regulate RANKL and OPG expressions. AGE also failed to stimulate RANKL and OPG promoter activities (Fig.S4C, D). Therefore, our results cue a new aspect of RAGE action in chondrocyte differentiation, and osteoclasts activation by RAGE would be independent on RANKL and OPG.

As described above, Cdxs came up as the candidates for RAGE stress indicator. Cdx1 was induced by RA transiently (Fig.6C). Although RA did not influence to cartilaginous matrix production or Ihh promoter activities without RAGE stress, RA responsiveness was observed only in stress condition (Fig.6A, B, E). Basal Cdx1 protein expression levels were clearly reduced in *RAGE*-transduced cells compared to GFP cells, and Cdx1 was induced in response to RA even in *RAGE*-transduced cells (Fig.6D). Therefore, we suggested that Cdx1 induction by RA may transiently work only in stress conditions such as excessive RAGE activation. We preliminarily examined Cdxs protein stability and intracellular localization using GFP-*Cdx1* transduced cells, and speculated from the data that RA treatment reduced Cdxs degradation hence increasing utilization efficiency of Cdxs. Further investigation will be required to clarify these observations and are currently in progress.

### Summary

In this study, we investigated the mechanisms of cartilaginous deterioration by RAGE, and found a pivotal role of Cdxs as the target of excessive RAGE activation state. Classically, anti-inflammation drugs such as NSAIDs or steroids are insufficient to control cartilaginous metabolic diseases such as osteoarthritis and rheumatoid arthritis. Deformation in metabolic cartilaginous disease remains a serious problem. We suggest the possibility of therapeutic effectiveness of RA in such conditions. We intend to proceed with further verification of RA effectiveness in the treatment of metabolic cartilaginous conditions with deformations.

## Supporting Information

Figure S1 **Cell cycle regulation by RAGE in ATDC5 and insufficiency of cell proliferation by AGE in MC3T3-E1.** (A–F) Each stable transfected cells at approximately 70% confluences in 145 mm dishes were fixed, and stained by propidium iodide according to manufactures instruction. Cell cycle analysis showed that *RAGE* reduces slightly G2/M phase, and increased G0/G1 phase compared to GFP. Furthermore, *RAGE* did not affect the cells in S phase. On the other hand, DN-*RAGE* had no effect on cell cycle. G, AGE did not stimulate cell proliferation in osteoblastic MC3T3-E1. n = 8–16.
(TIF)

Figure S2 **The Cdxs binding site in the mouse Ihh promoter region.** The red character indicates predicted Cdxs binding sites.
(TIF)

Figure S3 **HMGB1 inhibited RAGE-dependent chondrocytes differentiation.** A, HMGB1 inhibited cartilaginous matrix production. Indicated concentrations of HMGB1 were added 24 hours after plating. (B, C) RAGE dependency of HMGB1 action. B, Inhibited cartilaginous matrix production by HMGB1 was restored by DN-*RAGE*. C, NF-κB activation by HMGB1 was blocked by DN-*RAGE*. Respective adenoviruses were infected at approximately 50 MOI to established stable transfected cells of NF-κB-luc in ATDC5 cells. 24-h after infection, cells were treated with or without 1 µg/ml HMGB1 for 24-h, then cells were lyzed and analyzed NF-κB activities. Relative luciferase units (RLU) were shown. *P<0.05 vs GFP-Ad, †P<0.001 vs respective control (vehicle or HMGB1) in GFP-Ad. n = 8. Similar results were obtained from additional three experiments.
(TIF)

Figure S4 **AGE-RAGE did not regulate RANKL-OPG.** (A, B) RANKL or OPG mRNA did not regulated by RAGE. cDNA pools used were same as those used in Fig.2D-P. (C, D) AGE did not influenced RANKL or OPG promoter activities. 3-h after transfection of each constructs, ATDC5 cells were treated with non-glycated (BSA) or glycated BSA (AGE) at indicated concentrations. Respective activities were measured after 24 hours. There were no significant differences.
(TIF)

Figure S5 **RAGE expressed in chondrocytes both in** ***in vitro*** **and** ***in vivo*** **.** RAGE expression in primary chondrocytes (A), and in chondrogenic ATDC5 (B). (D, E) RAGE expression in cartilage.
(TIF)

Figure S6 **Cdx localization in fetal skeleton.**
(TIF)
