# Supplementary figures and images for: RAGE, Receptor of Advanced Glycation Endoproducts, Negatively Regulates Chondrocytes Differentiation
Source: PLoS One. 2014 Oct 2;9(10):e108819. doi: 10.1371/journal.pone.0108819 (PMC4183532; doi:10.1371/journal.pone.0108819)

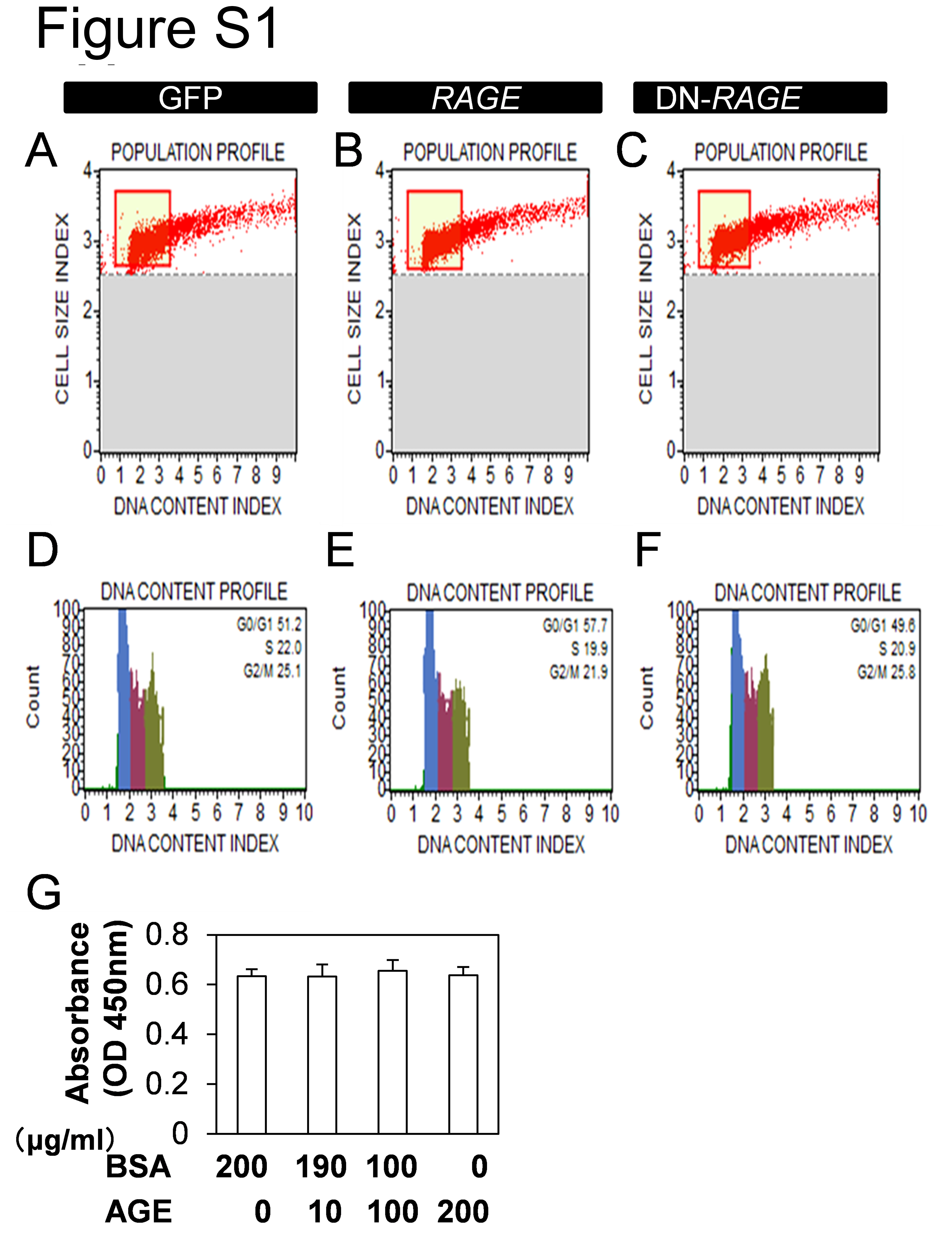

Supplement: Figure S1 — Cell cycle regulation by RAGE in ATDC5 and insufficiency of cell proliferation by AGE in MC3T3-E1. (A–F) Each stable transfected cells at approximately 70% confluences in 145 mm dishes were fixed, and stained by propidium iodide according to manufactures instruction. Cell cycle analysis showed that RAGE reduces slightly G2/M phase, and increased G0/G1 phase compared to GFP. Furthermore, RAGE did not affect the cells in S phase. On the other hand, DN-RAGE had no effect on cell cycle. G, AGE did not stimulate cell proliferation in osteoblastic MC3T3-E1. n = 8–16. (TIF) [file pone.0108819.s001.tif]

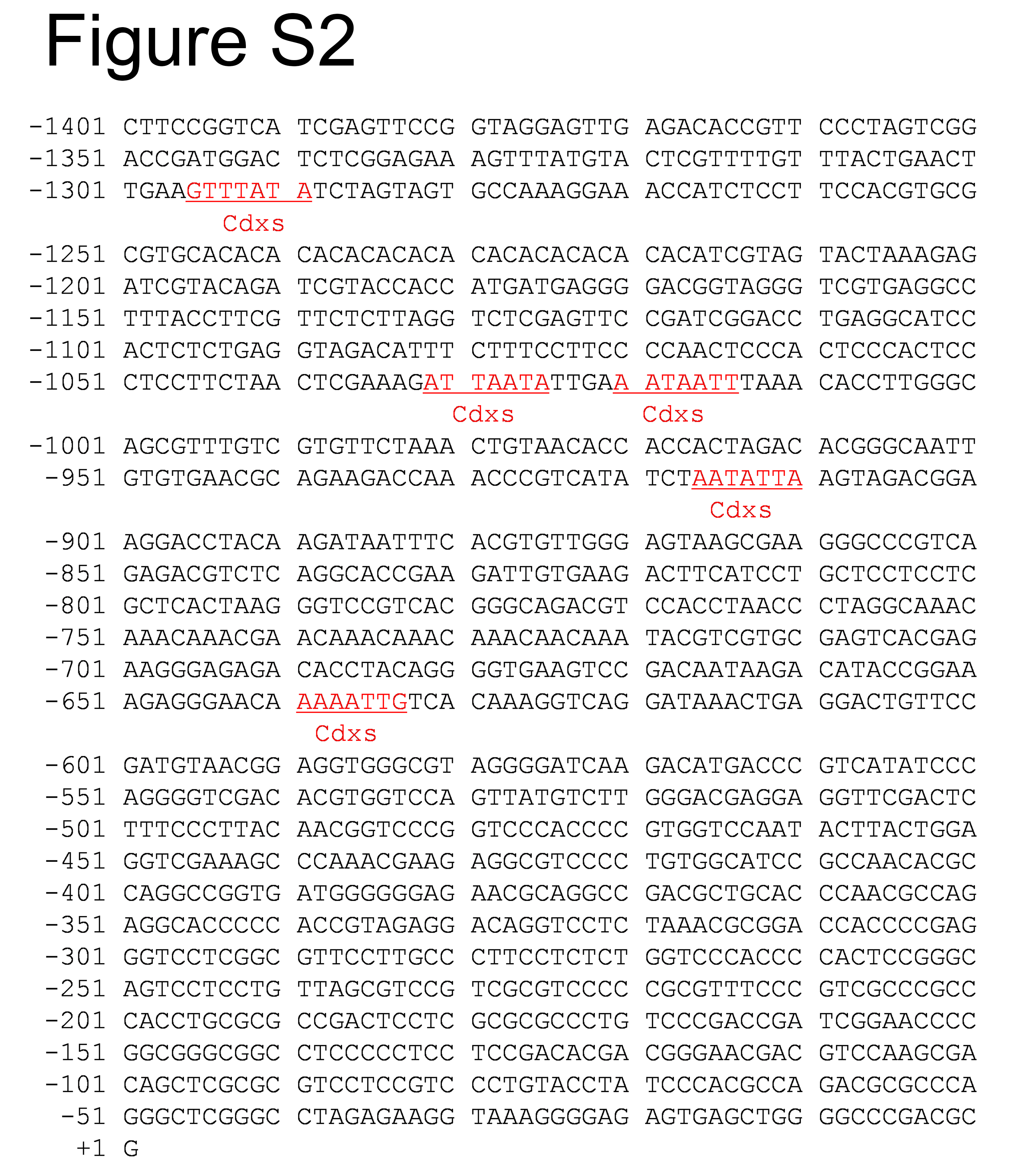

Supplement: Figure S2 — The Cdxs binding site in the mouse Ihh promoter region. The red character indicates predicted Cdxs binding sites. (TIF) [file pone.0108819.s002.tif]

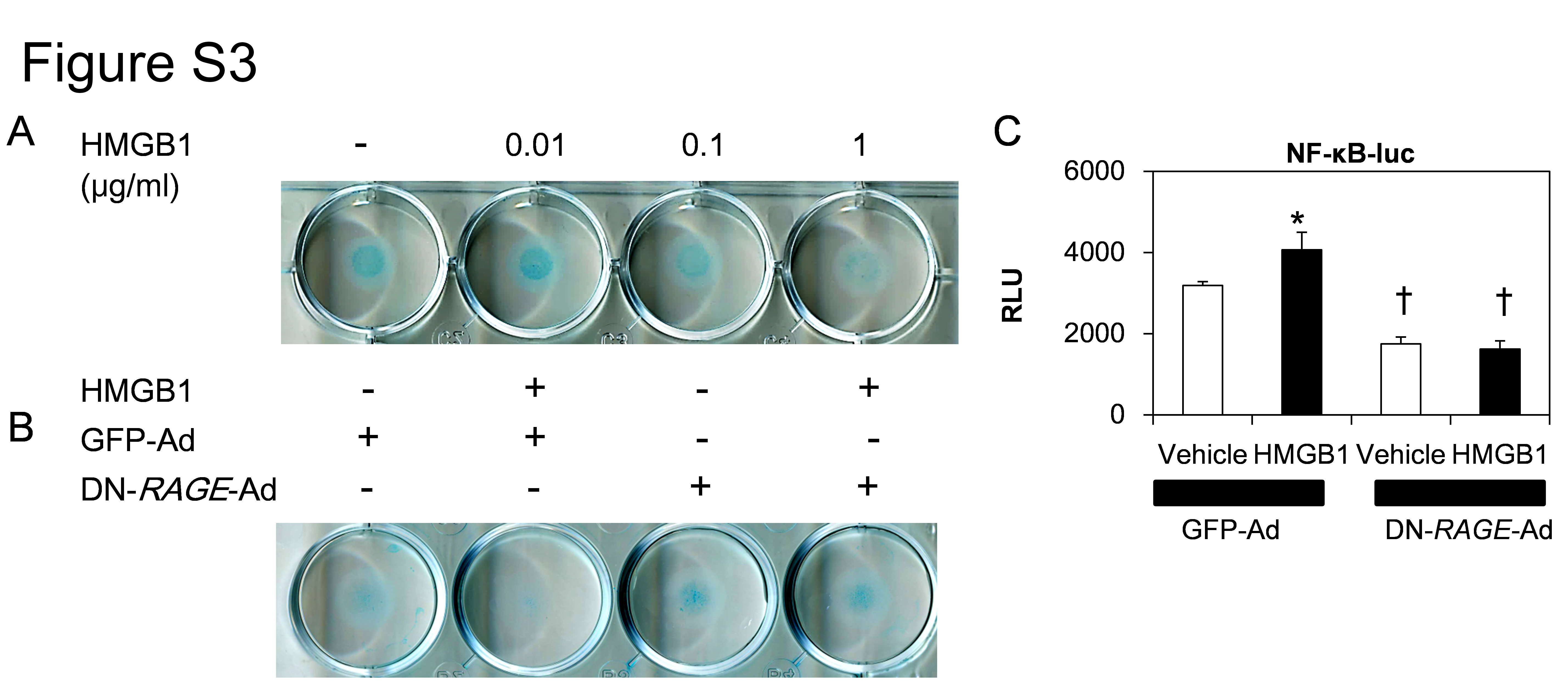

Supplement: Figure S3 — HMGB1 inhibited RAGE-dependent chondrocytes differentiation. A, HMGB1 inhibited cartilaginous matrix production. Indicated concentrations of HMGB1 were added 24 hours after plating. (B, C) RAGE dependency of HMGB1 action. B, Inhibited cartilaginous matrix production by HMGB1 was restored by DN-RAGE. C, NF-κB activation by HMGB1 was blocked by DN-RAGE. Respective adenoviruses were infected at approximately 50 MOI to established stable transfected cells of NF-κB-luc in ATDC5 cells. 24-h after infection, cells were treated with or without 1 µg/ml HMGB1 for 24-h, then cells were lyzed and analyzed NF-κB activities. Relative luciferase units (RLU) were shown. *P<0.05 vs GFP-Ad, †P<0.001 vs respective control (vehicle or HMGB1) in GFP-Ad. n = 8. Similar results were obtained from additional three experiments. (TIF) [file pone.0108819.s003.tif]

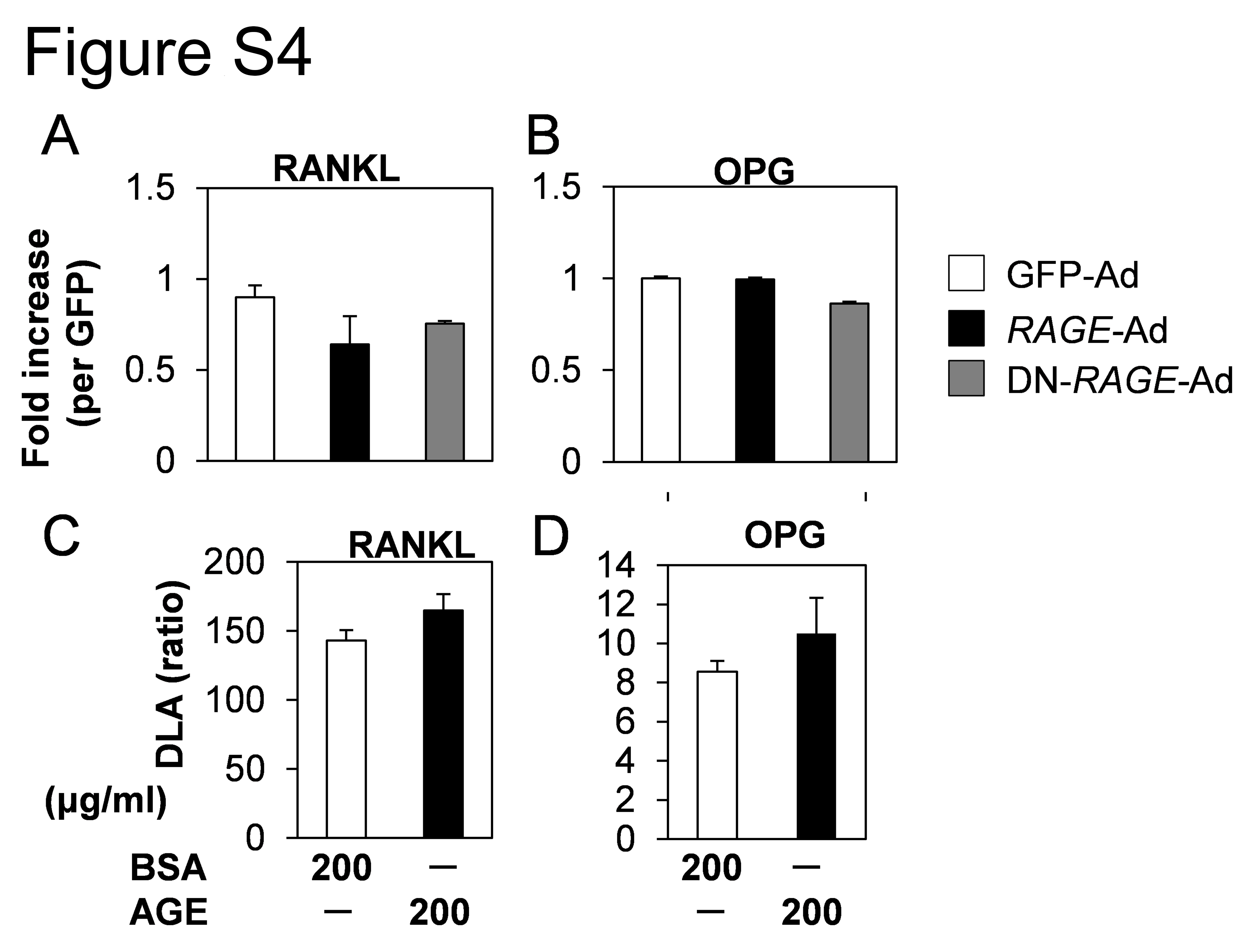

Supplement: Figure S4 — AGE-RAGE did not regulate RANKL-OPG. (A, B) RANKL or OPG mRNA did not regulated by RAGE. cDNA pools used were same as those used in Fig.2D-P. (C, D) AGE did not influenced RANKL or OPG promoter activities. 3-h after transfection of each constructs, ATDC5 cells were treated with non-glycated (BSA) or glycated BSA (AGE) at indicated concentrations. Respective activities were measured after 24 hours. There were no significant differences. (TIF) [file pone.0108819.s004.tif]

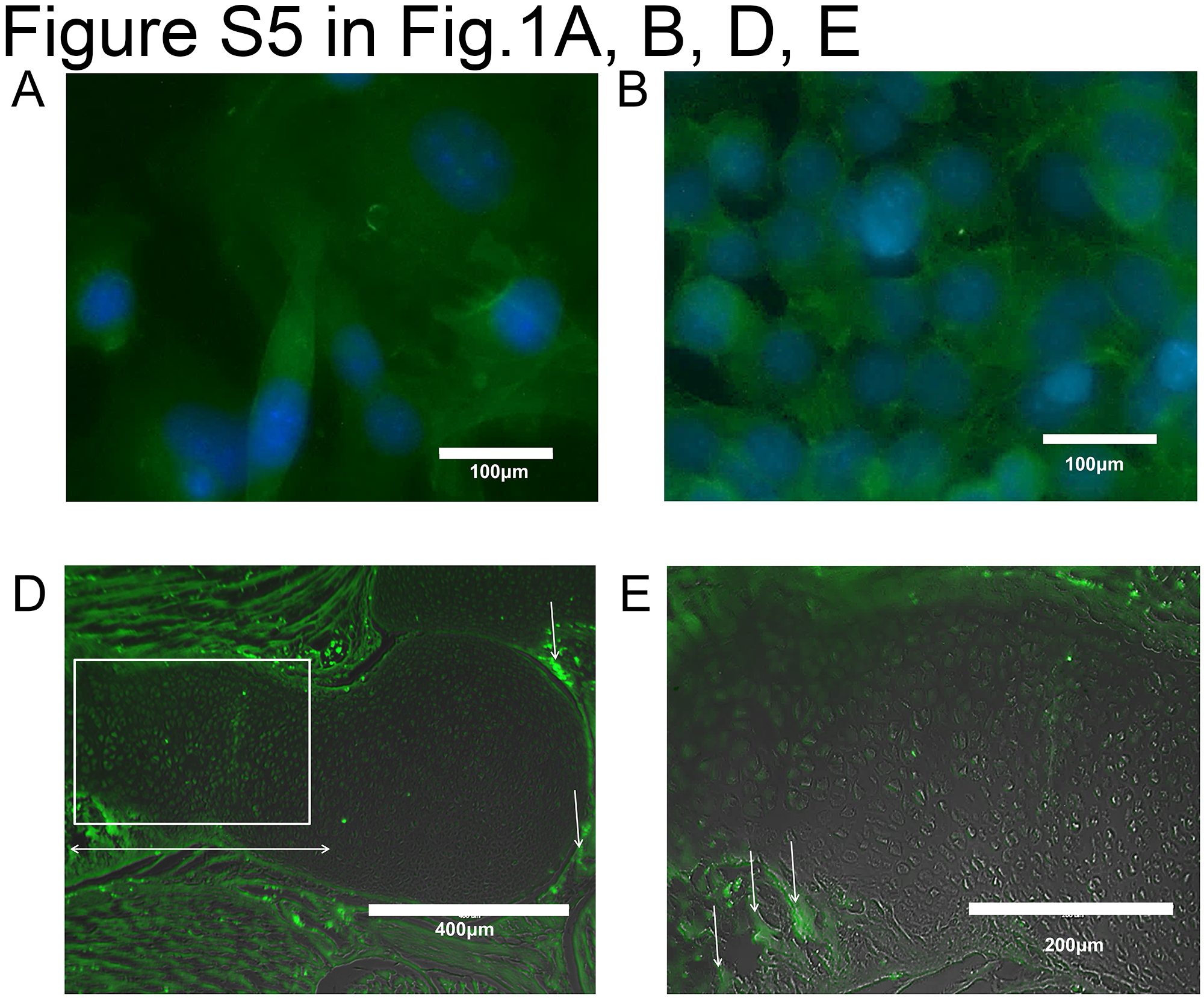

Supplement: Figure S5 — RAGE expressed in chondrocytes both in in vitro and in vivo . RAGE expression in primary chondrocytes (A), and in chondrogenic ATDC5 (B). (D, E) RAGE expression in cartilage. (TIF) [file pone.0108819.s005.tif]

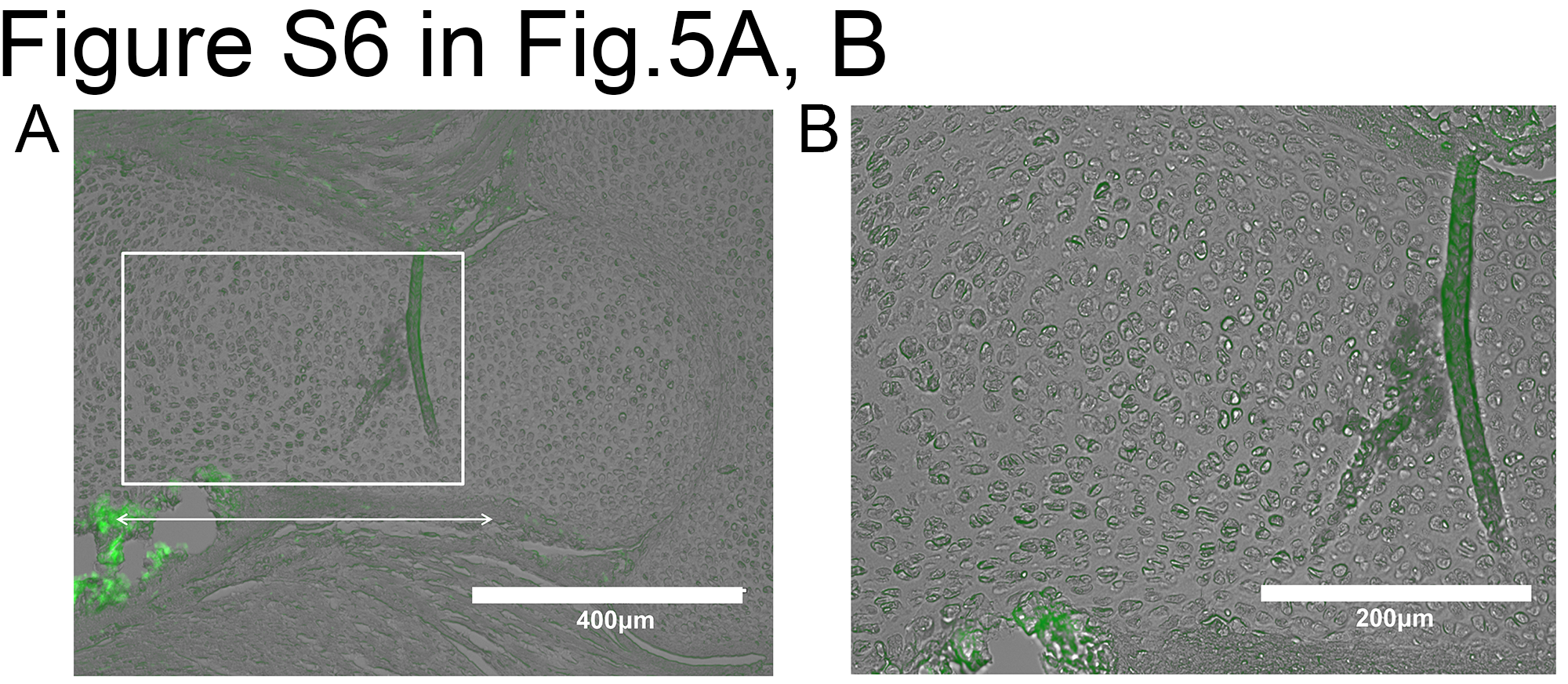

Supplement: Figure S6 — Cdx localization in fetal skeleton. (TIF) [file pone.0108819.s006.tif]
